# Pulmonary delivery of favipiravir inhalation solution for COVID-19 treatment: *in vitro* characterization, stability, *in vitro* cytotoxicity, and antiviral activity using real time cell analysis

**DOI:** 10.1080/10717544.2022.2118398

**Published:** 2022-09-05

**Authors:** Ayca Yildiz Pekoz, Ozlem Akbal Dagistan, Hanan Fael, Meltem Culha, Aybige Erturk, Nur Sena Basarir, Gokben Sahin, Muge Serhatli, Gamze Cakirca, Saban Tekin, Leyla Semiha Sen, Mustafa Sevim, Lutfiye Mulazimoglu Durmusoglu, Berrak C. Yegen

**Affiliations:** a Faculty of Pharmacy, Department of Pharmaceutical Technology, Istanbul University, Istanbul, Türkiye; b Faculty of Pharmacy, Department of Pharmaceutical Technology, Istinye University, Istanbul, Türkiye; c Faculty of Pharmacy, Department of Pharmaceutical Technology, Trakya University, Istanbul, Türkiye; d Medical Biotechnology (Marmara Research Center (MRC)), TUBITAK Marmara Research Center-MRC, Life Sciences, Kocaeli, Türkiye; e Molecular Biology and Genetics, Institute of Natural and Applied Sciences, Gebze Technical University, Kocaeli, Türkiye; f Hamidiye Faculty of Medicine, Department of Basic Medical Sciences, Medical Biology, University of Health Sciences, Istanbul, Türkiye; g School of Medicine, Basic Medical Sciences, Department of Physiology, Marmara University, Istanbul, Türkiye; h School of Medicine, Department of Infectious Diseases, Marmara University, Istanbul, Türkiye.

**Keywords:** Favipiravir, COVID-19, antiviral activity, inhaled formulation, respiratory

## Abstract

Favipiravir, an RNA-dependent RNA polymerase (RdRp) inhibitor, is used to treat patients infected with influenza virus and most recently with SARS-CoV-2. However, poor accumulation of favipiravir in lung tissue following oral administration has required an alternative method of administration that directly targets the lungs. In this study, an inhalation solution of favipiravir at a concentration of 2 mg mL^−1^ was developed and characterized for the first time. The chemical stability of inhaled favipiravir solution in two different media, phosphate buffer saline (PBS) and normal saline (NS), was investigated under different conditions: 5 ± 3 °C, 25 ± 2 °C/60% RH ± 5% RH, and 40 ± 2 °C/75% RH ± 5% RH; in addition to constant light exposure. As a result, favipiravir solution in PBS revealed superior stability over 12 months at 5 ± 3 °C. Antiviral activity of favipiravir was assessed at the concentrations between 0.25 and 3 mg mL^−1^ with real time cell analyzer on Vero-E6 that were infected with SARS-CoV-2/B.1.36. The optimum concentration was found to be 2 mg mL^−1^, where minimum toxicity and sufficient antiviral activity was observed. Furthermore, cell viability assay against Calu-3 lung epithelial cells confirmed the biocompatibility of favipiravir at concentrations up to 50 μM (7.855 mg mL^−1^). The *in vitro* aerodynamic profiles of the developed inhaled favipiravir formulation, when delivered with soft-mist inhaler indicated good lung targeting properties. These results suggest that favipiravir solution prepared with PBS could be considered as a suitable and promising inhalation formulation for pulmonary delivery in the treatment of patients with COVID-19.

## 1. Introduction

The pandemic of coronavirus disease 2019 (COVID-19) is caused by the severe acute respiratory syndrome coronavirus 2 (SARS-CoV-2) (Andersen et al., 2020; Zhu et al., 2021) infection, which may result in a wide range of symptoms from mild cases to pneumonia with life-threatening complications (Wiersinga et al., 2020). Favipiravir was one of the antivirals that was prescribed as a first line of treatment in some countries. It possesses a mechanism of an RNA-dependent RNA polymerase (RdRp) inhibitor that is expected to bind to, and inhibit RdRp, which prevents viral transcription and replication (Furuta et al., 2017; Shiraki & Daikoku, 2020). Favipiravir has been indicated for the treatment of influenza in Japan since 2014 (Shiraki & Daikoku, 2020). Promising results including accelerated discharge rate and less progression to mechanical ventilation were achieved by the favipiravir treatment in patients with mild and moderate COVID-19 (Almoosa et al., 2021; Alamer et al., 2021; Udwadia et al., 2021; Hassanipour et al., 2021). Various studies have demonstrated that the values of EC50 of Favipiravir against SARS-CoV-2 ranges from 62 µM to >500 µM (from 10 µg mL^−1^ to >78 µg mL^−1^) (Jeon et al., 2020; Shannon et al., 2020; Wang et al., 2020). Although favipiravir is generally well tolerated by the patients, changes in liver function parameters, hyperuricemia, and diarrhea are noted as common adverse effects (Ghasemnejad-Berenji & Pashapour, 2021). Studies have shown that patients with mild-to-moderate symptoms who were treated with favipiravir presented a significantly higher viral clearance rate, following 10 to 14 days after initiation of the treatment (Deng et al., 2022). The dosing regimen for patients with COVID-19 according to the Japanese Association for Infectious Diseases is 3600 mg (18 tablets/day) on the first day and 1600 mg (8 tablets/day) from second day onward, for up to 14 days (Joshi et al., 2021). However, a recent meta-analysis of clinical trials revealed no significant difference between the mortality rates of patients with COVID-19 treated with favipiravir and those not treated with favipiravir (Kow et al., 2022). Authors have underlined that the lack of beneficial effect of favipiravir treatment on mortality could be associated with the complex pharmacokinetic profile of the orally administered favipiravir, leading to low plasma concentrations (Kow et al., 2022). Accordingly, lung targeting through inhalation of favipiravir is not only expected to overcome the low bioavailability of oral administration, but also aims to achieve a higher drug concentration in the lung tissues. Direct delivery of favipiravir to the lungs preserves the drug from the first-pass metabolism (Du & Chen, 2020), reduces the systemic adverse effects and increases patient adherence to the treatment.

Drug solutions could be transferred as aerosol dosage form and delivered to the lungs via nebulizers or soft mist inhalers. However, the elevated risk of viral transmission via nebulizers and their low drug accumulation rate in the lungs has hindered their usage in COVID-19 (Pilcer & Amighi, 2010; Amirav & Newhouse, 2020). Unlike traditional nebulizers, soft mist inhalers provide a relatively high drug deposition in the lungs (Newman et al., 1998; Pitcairn et al., 2005). In addition, soft mist inhalers offer a safe and easy drug delivery method to the lungs in COVID-19 due to their closed mouthpiece and formulation-wise producibility (Ari, 2020).

Favipiravir solution for inhalation was introduced for the first time and was investigated in terms of antiviral activity, *in vitro* cytotoxicity, physicochemical properties (pH, viscosity, and osmolarity), aerodynamic particle size, stability, and quantification by high-performance liquid chromatography (HPLC). These studies are noteworthy in terms of developing an alternative dosage form to the oral forms available in the market for treatment of diseases caused by not only SARS-CoV-2, but also other airborne viruses.

## 2. Materials and method

### 2.1. Materials

Favipiravir (Jiangsu Hansyn Pharmaceutical Co., China) was kindly provided by Polifarma, Türkiye. PulmoSpray® was kindly donated by Resyca BV (Enschede, The Netherlands). Dulbecco’s phosphate buffered saline (referred to as PBS, pH 7.40) was purchased from PAN Biotech (Germany). Normal saline 0.9% for parenteral use was purchased from a local pharmacy. HPLC grades of methanol and acetonitrile, dipotassium hydrogen phosphate, and phosphoric acid was purchased from Merck (Germany). Deionized water (Milli-Q ultrapure water system, Millipore) was used for the preparation of all buffer solutions. All reagents used in cell culture analysis were obtained from Sigma-Aldrich (Germany).

### 2.2. Preparation of favipiravir solutions

Dose calculations of favipiravir solution (between 0.25 and 3 mg mL^−1^) for lung delivery were made according to available literature based on EC99 values obtained from Ebola virus (Du and Chen, 2020). Conversion to inhalation dose was performed by taking into account the oral bioavailability of favipiravir and the expected pulmonary deposition from the inhaler (Erelel et al., 2021). Two different media; normal saline (NS) and phosphate buffered saline (PBS) were used to prepare favipiravir solutions. The required amount of favipiravir was dissolved in these media separately. Following the mechanical mixing process (IKA®Vortex Genius3), solutions were filtered through 0.22 μm membrane for sterilization.

#### 2.2.1. Antiviral activity of favipiravir solutions

The viral stocks used in the current study, B.1.36 strain, were provided by the Ministry of Health, Directorate of Public Health. All viral studies were conducted at biosafety level 3 (BSL-3) laboratories at TUBİTAK, Marmara Research Center, Genetic Engineering and Biotechnology Institute in Gebze, Türkiye. Vero E6 (ATCC® CRL-1586TM, p no. 18) cell line was obtained from the American Type Culture Collection (ATCC) and cultured in a low-glucose Dulbecco’s modified Eagle’s medium (DMEM/LOW GLUCOSE, HyClone, Cat. no. SH30021.01) supplemented with 10% fetal bovine serum (FBS; HyClone, Cat. no. SV30160.03) and 1% penicillin/streptomycin (10,000 units mL^−1^) (Gibco) at 37 °C and 5% CO_2_ (Teng et al., 2013).

For this study, a high-throughput, quantitative Real Time Cell Analysis (RTCA) assay was performed using xCELLigence RTCA MP (Agilent Technologies, CA, USA) to determine favipiravir’s effects on the virus by quantifying the viral cytopathic effect and examining progression in infected cells. The xCELLigence RTCA MP system evaluates the effects of viruses on cells by measuring the changes in cell numbers through cell index (CI) and electronic impedance (Teng et al., 2013; Fang et al., 2011). Furthermore, the RTCA system allows simple detection and observation of cellular death due to both the viruses or the antiviral impact of medications (Teng et al., 2013; Fang et al., 2011). The suppression of SARS-CoV-2-induced cytopathic effect was categorized as either ‘completely’ or ‘partially’ antiviral. The test sample was considered ‘completely’ antiviral, if the SARS-CoV-2-induced cytopathic effect was inhibited at the highest concentration tested. On the other hand, it was considered ‘partially’ antiviral, if the effect was delayed and did not entirely inhibit the cytopathic effect of the highest concentration (Zost et al., 2020).

Vero E6 cells were seeded as 2.5 × 10^4^ cells per well into the sterile, disposable 96-well E-plate of the xCELLigence RTCA MP device. A 24-h cycle was followed so that the seeded cells could attach and proliferate to the E-plate. The favipiravir solution samples were prepared following the protocols specified in Section 2.2 and further treated with 3.5 × 10^5^ PFU mL^−1^ of B.1.36 variant of SARS-CoV-2 virus for 1 h at 37 °C and 5% CO_2_ in incubator. The media in the wells was removed, and the samples treated with the virus were added and incubated for 160 h at 37 °C and 5% CO_2_ (Durdagi et al., 2022; Taşlı et al., 2022). The untreated cells, negative control (state of no infection), contained only the cells, media, and 2% FBS. On the other hand, the positive control (state of infection) of the experiment contained viruses in the media with 2% FBS. Different concentrations of the solutions were analyzed to determine the protective dose of the favipiravir against SARS-CoV-2 in Vero E6 cells. The equipment converted electrical impedance to CI during the procedure at 15 min intervals. A rise in CI reflects cell viability/health, whereas a fall in CI reflects cell death/illness. Vero E6 cells exposed to the SARS-CoV-2 virus were expected to exhibit a decrease in CI values, whereas treatment with favipiravir solution in a certain ratio would show a significant increase in CI values. After the test, the collected data were evaluated using CI value plots of each well versus controls using Agilent Technologies’ RTCA Software Pro 2.6.0 (Basic) (Agilent Technologies Inc., Santa Clara, CA, USA.) (Teng et al., 2013; Charretier et al., 2018).

#### 2.2.2. Cell viability assay of favipiravir solutions in calu-3 cell line

Calu-3 cells were obtained from ATCC (ATCC® HTB-55™; p no. 30-36 used) and cultured in DMEM (p no. 41965 Gibco) supplemented with 5% fetal bovine serum (FBS, Sigma p no. 7524) and 1% antibiotic–antimycotic solution (Sigma, p no. A5955) in the cell culture flasks and incubated at 37 °C and 5% CO_2_. Calu-3 cells were seeded at a density of 1 × 10^4^ cells/well in the 96-well plates and incubated at 37 °C and 5% CO_2_ for 24 h. Afterward, cells were treated with different concentration of favipiravir (1, 5 10, 25, and 50 µg mL^−1^). Following 24-h of incubation, 30 µL of 3-(4,5-dimethylthiazol-2-yl)-2,5-diphenyltetrazolium bromide (MTT) (Sigma, p no. M5655) solution (5 mg mL^−1^ in PBS) was added to each well, then the plates were incubated at 37 °C for 4 h. At the end of the incubation period, formazan crystals were dissolved by adding 100 µL of DMSO (Sigma, p no. D8418) into each well. The optical density was measured at 570 nm wavelength with BioTek Microplate reader (ELX50, Vermont, USA).

#### 2.2.3. Physicochemical characterization of favipiravir solutions

The pH (HI2002, Hanna Instruments, Germany), viscosity (Rheostress1, Thermo Scientific Haake Mars, Waltham, MA, USA), and osmolarity (Model 2020, Advanced Instruments, USA) of favipiravir solutions (2 mg mL^−1^) was examined in PBS and NS.

#### 2.2.4. Aerodynamic particle size analysis of favipiravir solutions

The Next Generation Impactor (NGI) system, consisting of a High-Capacity Pump (Model HCP5), Air Flow Meter (Model DFM 2000), Universal Induction Port (UIP, throat), and the NGI-170 body was used. The NGI setup and procedures as described in Inhaler Testing Guide and European Pharmacopeia (European Directorate for the Quality of Medicines and Healthcare [EDQM], 2009) were thoroughly followed for the soft-mist inhaler device (PulmoSpray®). The NGI aerosol parameters evaluated for favipiravir solution were the fine particle fraction (FPF), mass median aerodynamic diameter (MMAD), and geometric standard deviation (GSD). The collection surfaces of the NGI cups were coated with propylene glycol, as it was recommended by the European Pharmaceutical Aerosol Group. The whole NGI setup was cooled at 5 °C and examined before each inhaler run. A steady inhalation flow of 20 L min^−1^ (±5%) was drawn through the NGI configuration for a duration of 3 s per puff. A total of 1 mL sample was sprayed into the NGI. To recover the favipiravir deposited throughout the NGI, the mouthpiece adapter and throat were rinsed thoroughly with a mixture of methanol–water (1:4). The amounts deposited on each collection cup along with the mouth and throat were determined by HPLC (see Section 2.2.6.), and the results were analyzed by C.I.T.D.A.S. program.

#### 2.2.5. Stability study of favipiravir solutions

The stability of favipiravir solution (2 mg mL^−1^) in two different media, PBS and NS, was investigated. Each solution was aliquoted and transferred into sealed glass vials. Some of the vials were exposed to constant light exposure for photostability assessment. Other vials were protected from light by wrapping with aluminum foil and kept at three different conditions: 5 ± 3 °C, 25 ± 2 °C/60% RH ± 5% RH, and 40 ± 2 °C/75% RH ± 5% RH for 12 months. The chemical stability of favipiravir solutions was assessed at week 1, 2, 3, and 4 and the following months (2, 3, 6, 9, and 12 months) from initial date. Tests were evaluated according to the ICH Q1A and ICH Q1B guidelines and Pharmacopeial test limits were applied.

#### 2.2.6. Quantification of favipiravir by HPLC

A stability indicating reversed phase HPLC method was developed and validated for the quantification of favipiravir. An HPLC system (Shimadzu, Japan) equipped with photodiode array detector, autosampler, quaternary pump, and column oven was utilized. The analysis was performed using C18 column (250 mm × 4.6 mm, 5 μm) at 30 °C. The mobile phase consisted of 80% of solution A (50 mM of potassium dihydrogen phosphate was adjusted with sodium hydroxide to pH 6.8) and 20% of solution B (acetonitrile:methanol, 1:1 vol/vol). The flow rate was 1 mL min^−1^, the injection volume was 10 μL and the detection wavelength was 364 nm. The method was validated for linearity, specificity, precision, and accuracy over the concentration range of 5–100 μg mL^−1^.

### 2.3. Statistical analysis

All statistical analyses were performed using GraphPad Prism 9.4.1 (GraphPad Software, San Diego, CA, USA). Data are presented as mean ± standard error. One-way analysis of variance (one-way ANOVA) and then Bonferroni post hoc comparison test were conducted for comparisons between groups. *p* < 0.001 value was considered significant.

## 3. Results

### 3.1. Antiviral activity test

The B.1.36 coding variant form of the SARS-CoV-2 virus was studied to assess the antiviral activity of favipiravir through Vero E6 cells within doses ranging from 0.5 mg mL^–1^ to 3 mg mL^–1^; this concentration range was determined as a result of favipiravir’s cytotoxicity results (data not shown). Data were gathered at intervals of 15 min over 160 h and was expressed as Normalized cell index (NCI) units. The data shown in the figures are normalized according to the time point when the virus was added to the experiment.

The following observation was made during the study period:

- The virus was introduced to the cells on the 24th hour
- Cell death did not occur until the 50th hour, and by the 114th hour there were no viable cells left
- The cells that received no virus (control) were viable for 160 h. However, the growth curve dropped significantly in the late hours due to nutrient deficiency (Zost et al., 2020; Taşlı et al., 2022).
- The NCI data ranged from 1% at 0.25 mg mL^−1^, 27% at 0.5 mg mL^−1^, to 100–117% at 1–3 mg mL^−1^. NCI values obtained from these concentration ranges were either comparable to or higher than the negative control.
- Extended data Figure 1 illustrates representative graphs for the complete and partial antiviral samples.

In conclusion, it was observed that the favipiravir solution displays full antiviral efficacy against SARS-CoV-2/B.1.36 at 1–3 mg mL^−1^ concentrations.

**Figure 1.**
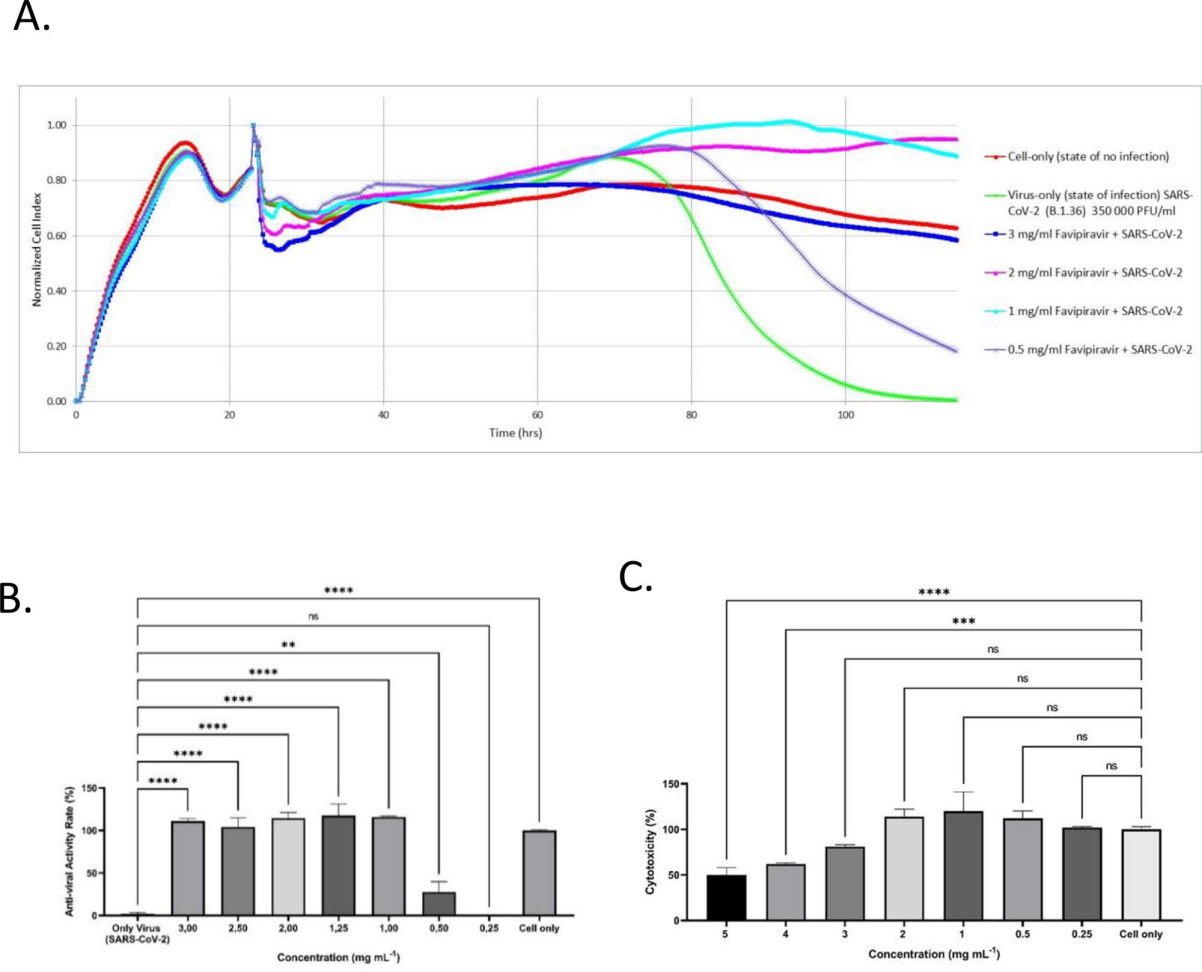
Anti-viral efficacy of favipiravir molecule using xCELLigence RTCA MP real-time cell analysis device. Quantifying favipiravir molecule neutralizing titer using Agilent xCELLigence RTCA. A: Vero E6 cells were infected with 3.5 × 10^5^ PFU mL^−1^ of SARS-CoV-2 (B1.36), that were pre-incubated with different concentrations of favipiravir molecule. Cell only, Vero E6 cells that were not infected with the virus (red line); Only-Virus, Vero E6 cells infected with the virus that was not pre-exposed to favipiravir molecule (green line). B: The anti-viral rate bar graph with standard deviation. C: The cytotoxicity rate bar graph of favipiravir molecule on Vero E6 cells with standard deviation. The data shown in the figure was normalized according to the time point where the virus was added to the experiment. In (A) and (B) (****) indicate *p* < 0.001 by one-way ANOVA with Dunnet’s multiple comparisons.

### 3.2. Cell viability assay of favipiravir in calu-3 cell line

**Figure 2.**
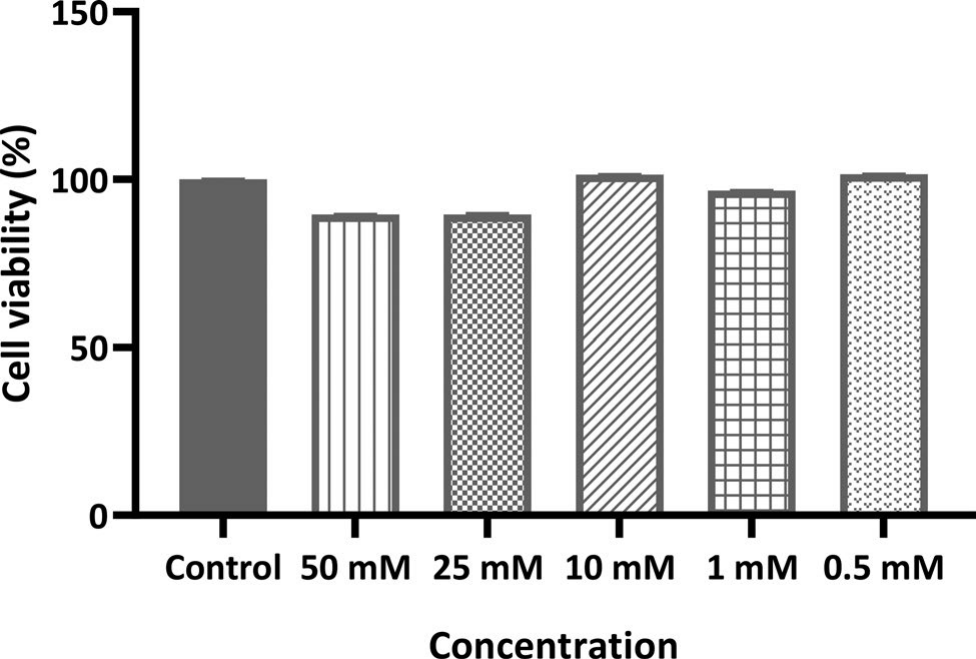
Effects of favipiravir on the viability of Calu-3 lung epithelial cells at different concentrations (50–0.1 mM which is 0.1571–7.855 mg mL^−1^, respectively) as measured by the MTT assay. Values are expressed as mean ± SD (*n* = 6).

The cytotoxicity of favipiravir solution prepared in normal saline was evaluated *in vitro* for Calu-3 lung epithelial cells, and there was no significant statistically difference in cell viability levels within the range of 50–0.1 mM (which corresponds to 0.1571–7.855 mg mL^−1^ respectively) of favipiravir compared with the control group cells (favipiravir solution was not applied to the control group) (Figure 2).

### 3.3. Physicochemical characterization of favipiravir solutions

Favipiravir solutions were prepared at 2 mg mL^−1^ in both NS and PBS. Due to the acidic nature of favipiravir, the pH of its solution in NS was found to be 3.46. However, the pH increased to 5.26, when favipiravir solution was prepared in PBS. It was observed that the viscosity values of favipiravir solutions are within the range of 1.009–1.104 mPa.s, and they showed similar viscosity properties. Osmolality of the prepared favipiravir solutions in two different medias were 299 mOsm kg^−1^ in PBS and 303 mOsm kg^−1^ in NS, which were significantly similar to each other.

### 3.4. Aerodynamic particle size characterization of favipiravir solutions

Aerodynamic particle size distribution data are interpreted with MMAD, GSD, and FPF (percentage of particles with aerodynamic particle size below 5 µm). These values for favipiravir solution in NS and PBS are given in Table 1. Both formulations show no significant difference in terms of aerodynamic particle size distribution.

**Table 1.** Particle size distribution obtained for favipiravir solutions.

Parameter	Favipiravir in NS	Favipiravir in PBS
MMAD^a^ (µm)	5.43 ± 0.32	4.83 ± 0.11
FPF 5 µm^b^ (%)	45.63 ± 2.43	52.66 ± 1.67
GSD^c^	1.63 ± 0.07	1.70 ± 0.01

a Mean mass aerodynamic diameter.

b Fine particle fraction.

c Geometric standard deviation (mean ± SD; *n* = 3).

**Figure 3.**
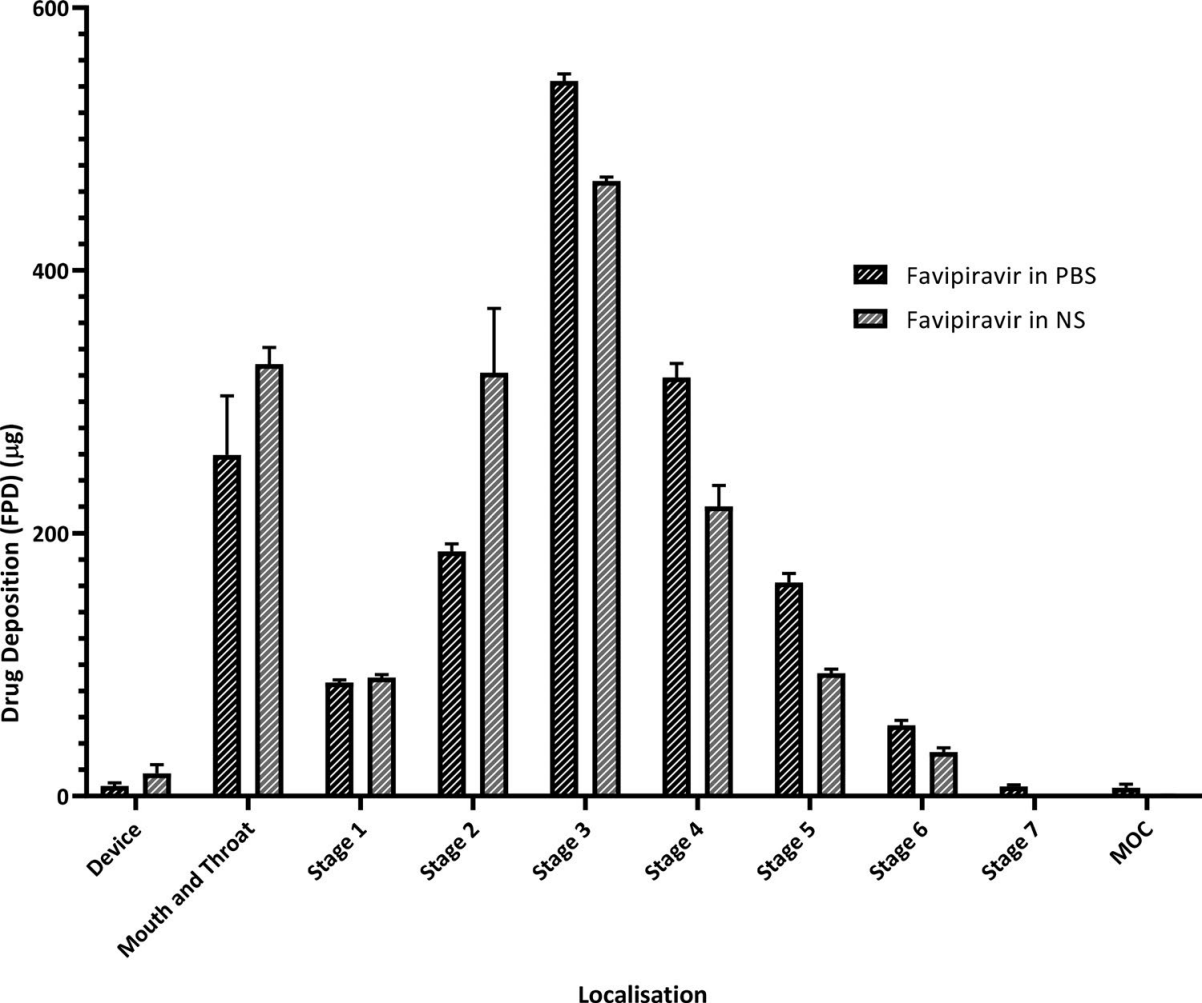
Drug deposition profile of favipiravir solutions in NGI stages. Values are expressed as mean ± SD (*n* = 6).

The deposition of favipiravir solutions on each stage of NGI compartments is shown in Figure 3. Particles deposited on each stage were given as the amount of fine particle dose (µg).

### 3.5. Stability study of favipiravir solution

**Figure 4.**
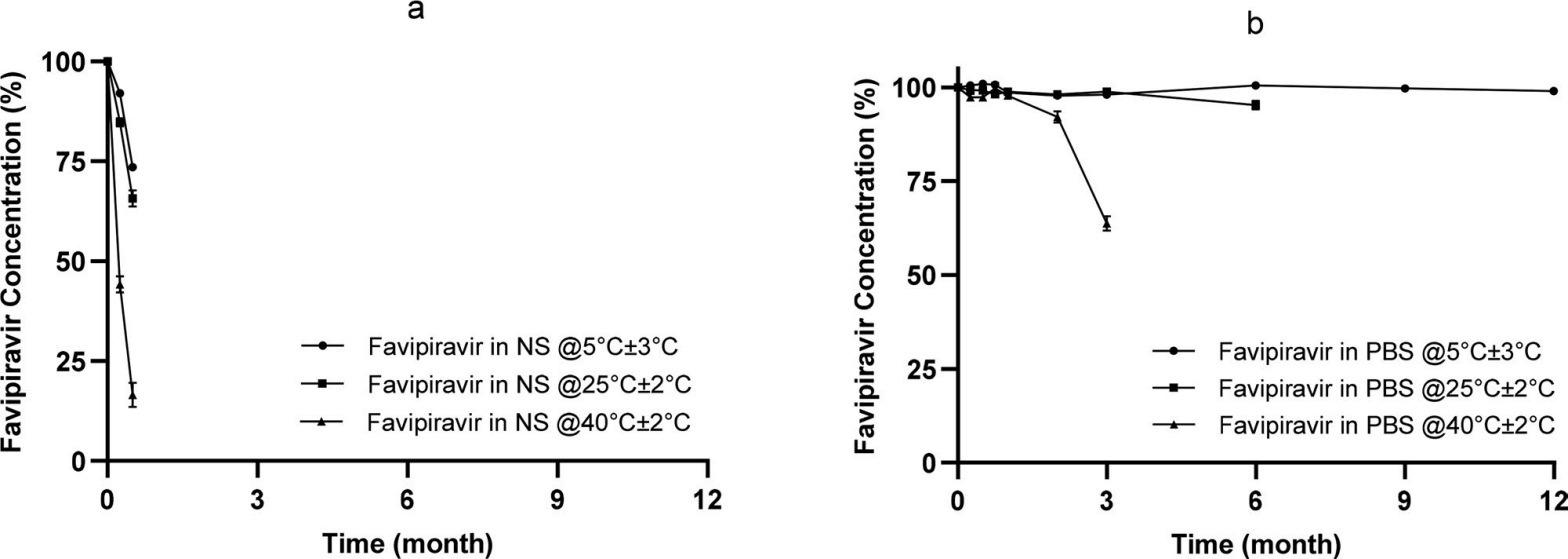
Stability profiles of favipiravir in (a) normal saline and (b) PBS, stored protected from light at 5C ± 3 °C, 25 ± 2 °C, and 40 ± 2 °C [Values are expressed as mean ± SD (*n* = 3), note that the error bars at some points are of the size of the symbols].

The stability of favipiravir solutions in NS and PBS was investigated under three different storage conditions (5 ± 3 °C, 25 ± 2 °C/60%RH ± 5%RH, and 40 ± 2 °C/75%RH ± 5% RH). In addition, the photostability of the solutions was assessed under constant light exposure. Stability data revealed that favipiravir solution prepared in PBS showed superior stability over that prepared in NS. Favipiravir solution in PBS remained stable throughout 12 months of storage at 5 °C (favipiravir assay at 12 months; 100.50 ± 0.71%), while it was stable up to 3 months (97.20 ± 1.03%) and 1 month (97.80 ± 1.10%) at 25 °C and 40 °C, respectively (Figure 4). On the contrary, favipiravir solution in NS retained its stability up to 1 week at 5 °C (95.60 ± 0.90%), while the concentration dramatically dropped to ∼84.4% and ∼44.2% after 1 week at 25 °C and 40 °C, respectively. Furthermore, light exposure has accelerated the degradation of favipiravir in both solutions. After one week of exposure to constant illumination, the concentration of favipiravir decreased to ∼88.8% and ∼81.6% in NS and PBS, respectively.

## 4. Discussion

The COVID-19 pandemic has underlined the importance of inhaled therapy due to the inadequacy of conventional treatments. Although oral drugs are generally preferred by patients, alternative dosage forms are crucial in terms of drug accumulation in the target organ. Studies have shown that airborne transmission is the dominant route for the spread of SARS-CoV-2, the causative virus of COVID-19 (Zhang et al., 2020), and most infections cause mild to moderate illness with respiratory symptoms (Elezkurtaj et al., 2021). Yet even though the virus is a respiratory one, there are no inhaled antivirals available for the treatment of the disease. Therefore, the aim of this study was to develop and characterize an inhaler dosage form for the treatment of COVID-19.

Scientists have taken a repurposing approach for the treatment of COVID-19, and thus lists of antiviral candidates were re-investigated. Examples of these antivirals are remdesivir, lopinavir-ritonavir, umifenovir, favipiravir, chloroquine-hydroxychloroquine, and molnupiravir (Sahin et al., 2022). However, no antiviral drug has been successful in the clinical treatment of COVID-19, although their efficacy was shown *in vitro* against SARS-CoV-2. In a study, it was reported that the reason oral Lopinavir could not be beneficial in patients with COVID-19 may be due to the insufficient concentration level of the drug in the lungs (Wang & Chen, 2020), a hypothesis that was based on a tissue distribution study of orally administered Lopinavir-Ritonavir in rats (Kumar et al., 2004).

Favipiravir shows antiviral activity against many RNA viruses, making it a promising drug for the treatment of many infections associated with RNA viruses (Furuta et al., 2017), including COVID-19. In a biodistribution study of [^18^F] Favipiravir in mice, it has been shown that the antiviral agent accumulates in the lungs at a very low ratio. This strengthens the hypothesis that an effective treatment cannot be achieved in COVID-19 through conventional dosage use of antivirals. An effective dose of drug in the lungs can be achieved by inhalation treatment and the determination of this appropriate dose is a crucial factor. Favipiravir should have the least toxic effect on the body, while effectively preventing SARS-CoV-2 viruses. Therefore, a system, designed for real-time monitoring of *in vitro* cellular adhesion properties using E-plates called the xCELLigence RTCA was used in this study. RTCA is more durable, needs less manual affirmation, saves time, and does not require labels like traditional methods (Teng et al., 2013) [the viral plaque test, the median tissue culture infective dose (TCID50), and detection of presence by PCR and RT-PCR] for the investigation of viruses. In this study, the Vero E6 cell line was chosen for investigation, replication, and isolation of SARS-CoV-2. These cells are distinguished by the fact that they do not produce interferon (IFN), and IFN deficiency is essential for the replication of SARS-CoV-2. In addition to IFN insufficiency, the cells exhibit high ACE-2 expression on their membrane surface. Furthermore, this cell line produces a high load of virus particles (Takayama, 2020). The study period lasted for 160 h, in which the cells were observed and assessed at 15-min intervals. The final data has shown favipiravir was not cytotoxic to the Vero E6 cell line at concentrations between 0.25 and 3 mg mL^−1^. Furthermore, cytotoxicity studies were carried out on Calu-3 cells confirmed the safety of favipiravir within the concentration range of 0.1571–7.855 mg mL^−1^.

Due to the acidic nature of the molecule, the pH of favipiravir solution in NS was found to be ∼3.46. However, the pH was ∼5.26 when favipiravir solution was prepared in PBS. Given that the pH of the lung fluid is around 6.6 in healthy individuals (Fischer & Widdicombe, 2006), favipiravir solution in PBS appears suitable for administration to the lungs. Osmolality values of favipiravir solutions (2 mg mL^−1^) were quite similar to each other, 299 mOsm kg^−1^ in PBS and 303 mOsm kg^−1^ in NS. Also, they are both compatible with the physiological value (∼288 mOsm kg^–1^) (Fazekas et al., 2013). Furthermore, favipiravir solutions were found to have similar viscosity properties, with viscosity values in the range of 1.009–1.104 mPa s.

When the subject comes to inhalation technology, another factor to consider is the choice of inhalation devices (e.g., inhaler). Choosing an appropriate device affects parameters such as patient compliance, stability, and drug accumulation. Soft-mist inhalers are a relatively new class of ‘non-pressurised metered dose inhalers’. According to the available literature, soft-mist inhalers provides a higher drug accumulation in the lungs than most pressurized metered dose inhalers, dry powder inhalers, and nebulizers. Soft-mist inhalers owes this advantage to the fact that it produces an aerosol with a larger fraction of fine particles than the other devices, and the aerosol produced leaves the inhaler at a slower pace and lasts longer (Pitcairn et al., 2005; Anderson, 2006; Brand et al., 2008; Leiner et al., 2017). A Phase IIb clinical trial has recently been conducted by our team in which low molecular weight heparin was administered to patients with severe COVID-19 via a soft-mist inhaler. At the end of the study, the drug was well tolerated and significantly decreased the need for oxygen therapy (Erelel et al., 2021).

Aerodynamic particle size distribution (APSD) is a crucial parameter for lung delivery, which is a virtual measurement of the diameter of a particle in the airflow. It is characterized by the MMAD and the GSD. For local effect, the desirable target point of the drug particles is to the bronchi and bronchioles. This requires a particle aerodynamic size within the range of 2–5 µm. Whereas, for systemic effect via inhalation, smaller particles (approximately 1–3 μm) are required. Therefore, APSD is used to predict the region and amount of an active ingredient deposited in the pulmonary area and this parameter is consider as a ‘critical quality’ attribute for the development of inhalation formulations. Consistent APSD values were obtained for both favipiravir solutions through the study period. Both formulations delivered via soft-mist inhaler similarly accumulated in stages 2–8 of impactor, which corresponds to a cutoff diameter of 7.613–0.77 µm. This data shows that the favipiravir solution for inhalation is within the appropriate range of droplet size required to target the bronchi and bronchioles when administered via the soft-mist inhaler. The MMAD values of favipiravir solution in NS and PBS were 5.43 ± 0.32 µm and 4.83 ± 0.11 µm, respectively, and the FPF 5 µm values were 45.63 ± 2.43% and 52.66 ± 1.67%, respectively (Table 1). There was no significant difference in the APSD values between the two formulations. Thus, both formulations were considered appropriate for local lung targeting (Ciciliani et al., 2021). Furthermore, they both show high FPF values, which indicate that the aerodynamic particle sizes are below 5 µm. In turn, this value represents a crucial point for assessment of lung localization, especially in the targeted area.

The stability of a pharmaceutical solution is essential to its efficacy, thus favipiravir solutions were kept at different storage conditions including 5C ± 3 °C, 25 ± 2 °C/60% RH ± 5% RH, and 40 ± 2 °C/75% RH ± 5% RH. Since the highest stability of favipiravir solution was obtained at 5 ± 3 °C, this condition was considered to simulate long-term conditions. Consequently, storage conditions of both 25 ± 2 °C/60% RH ± 5% RH and 40 ± 2 °C/75% RH ± 5% RH were considered as accelerated conditions in our study. Stability data have revealed the dependence of favipiravir degradation on the pH rather than temperature, as degradation in normal saline (∼pH 3.46) was significant at all studied different temperatures (Figure 4a). Upon utilization of PBS, the pH of favipiravir solution increased to ∼5.26 that greatly enhanced its stability up to one year at 5 ± 3 °C. However, the poor stability of favipiravir in NS (pH 3.46) is consistent with recent studies that showed the sensitivity of favipiravir toward acidic, basic, and oxidative conditions (Marzouk et al., 2022; Vemuri et al., 2022). At this acidic pH, one of degradation routs might be the cleavage of amide group in favipiravir molecule which was proposed by Vemuri et al. (2022).

## 5. Conclusion

To date, oral favipiravir has been used in many countries to treat patients infected with SARS-CoV-2. However, current literature points out that oral favipiravir has a poor accumulation in the lungs, which causes ineffective treatment. In this study, an inhaled formulation of favipiravir was developed for the first time to provide an effective treatment in the lungs. In the *in vitro* antiviral activity study, it was found that a concentration of 2 mg mL^−1^ of favipiravir provides an effective antiviral activity on SARS-CoV-2. Considering the high doses (1600 mg–3600 mg) that were prescribed orally, the fact that a small dose such as 2 mg mL^−1^ can achieve an antiviral efficacy in the lungs is an indication of the superiority of an inhalation formulation. There is an ongoing preclinical study with the inhaled favipiravir formulation developed by our group. We hope that this study will pave the way for more clinical trials in the treatment of COVID-19 using inhalation formulations.

## Declaration of competing interest

The authors declare that they have no known competing financial interests or personal relationships that could have appeared to influence the work reported in this paper.

## References

[CIT0001] Alamer A, Alrashed AA, Alfaifi M, et al. (2021). Effectiveness and safety of favipiravir compared to supportive care in moderately to critically ill COVID-19 patients: a retrospective study with propensity score matching sensitivity analysis. Curr Med Res Opin 37:1085–97. 33890544 10.1080/03007995.2021.1920900 PMC8146299

[CIT0002] Almoosa Z, Saad M, Qara S, et al. (2021). Favipiravir versus standard of care in patients with severe COVID-19 infections: a retrospective comparative study. J Infect Public Health 14:1247–53. 34464921 10.1016/j.jiph.2021.08.022 PMC8382493

[CIT0003] Amirav I, Newhouse MT. (2020). Transmission of coronavirus by nebulizer: a serious, underappreciated risk. CMAJ 192:E346. 32392488 10.1503/cmaj.75066 PMC7124163

[CIT0004] Andersen KG, Rambaut A, Lipkin WI, et al. (2020). The proximal origin of SARS-CoV-2. Nat Med 26:450–2. 32284615 10.1038/s41591-020-0820-9 PMC7095063

[CIT0005] Anderson P. (2006). Use of Respimat Soft Mist inhaler in COPD patients. Int J Chron Obstruct Pulmon Dis 1:251–9. 18046862 10.2147/copd.2006.1.3.251 PMC2707154

[CIT0006] Ari A. (2020). Promoting safe and effective use of aerosol devices in Covid-19: risks and suggestions for viral transmission. Expert Opin Drug Deliv 17:1509–13. 32799579 10.1080/17425247.2020.1811225

[CIT0007] Brand P, Hederer B, Austen G, et al. (2008). Higher lung deposition with Respimat® Soft Mist^TM^ Inhaler than HFA-MDI in COPD patients with poor technique. Int J COPD 3:763–70. PMC2650591 19281091

[CIT0008] Charretier C, Saulnier A, Benair L, et al. (2018). Robust real-time cell analysis method for determining viral infectious titers during development of a viral vaccine production process. J Virol Methods 252:57–64. 29154792 10.1016/j.jviromet.2017.11.002

[CIT0009] Ciciliani AM, Denny M, Langguth P, et al. (2021). Lung deposition using the Respimat® Soft Mist^TM^ inhaler mono and fixed-dose combination therapies: an in vitro/in silico analysis. COPD J Chronic Obstr Pulm Dis 18:91–100. 10.1080/15412555.2020.1853091 33302718

[CIT0010] Deng W, Yang C, Yang S, et al. (2022). Evaluation of favipiravir in the treatment of COVID-19 based on the real-world. Expert Rev anti Infect Ther 20:555–65. 34846960 10.1080/14787210.2022.2012155 PMC8787837

[CIT0011] Du Y, Chen X. (2020). Favipiravir: pharmacokinetics and concerns about clinical trials for 2019-nCoV infection. Clin Pharmacol Ther 108:242–7. 32246834 10.1002/cpt.1844

[CIT0012] Durdagi S, Avsar T, Orhan MD, et al. (2022). The neutralization effect of montelukast on SARS-CoV-2 is shown by multiscale in silico simulations and combined in vitro studies. Mol Ther 30:963–74. 34678509 10.1016/j.ymthe.2021.10.014 PMC8524809

[CIT0013] Elezkurtaj S, Greuel S, Ihlow J, et al. (2021). Causes of death and comorbidities in hospitalized patients with COVID-19. Nat Sci Rep 11:4263. 10.1038/s41598-021-82862-5 PMC7895917 33608563

[CIT0014] Erelel M, Kaskal M, Akbal-Dagistan O, et al. (2021). Early effects of low molecular weight heparin therapy with soft-mist inhaler for COVID-19-induced hypoxemia: a phase IIb trial. Pharmaceutics 13:1768–13. 34834183 10.3390/pharmaceutics13111768 PMC8618458

[CIT0015] European Directorate for the Quality of Medicines and Healthcare (EDQM). (2009). Section 2.9.18—Preparations for inhalation: aerodynamic assessment of fine particles. European Pharmacopeia. Strasbourg, France: Council of Europe, 287–300.

[CIT0016] Fang Y, Ye P, Wang X, et al. (2011). Real-time monitoring of flavivirus induced cytopathogenesis using cell electric impedance technology. J Virol Methods 173:251–8. 21349291 10.1016/j.jviromet.2011.02.013 PMC3086694

[CIT0017] Fazekas AS, Funk G-C, Klobassa DS, et al. (2013). Evaluation of 36 formulas for calculating plasma osmolality. Intensive Care Med 39:302–8. 23081685 10.1007/s00134-012-2691-0

[CIT0018] Fischer H, Widdicombe JH. (2006). Mechanisms of acid and base secretion by the airway epithelium. J Membr Biol 211:139–50. 17091214 10.1007/s00232-006-0861-0 PMC2929530

[CIT0019] Furuta Y, Komeno T, Nakamura T. (2017). Favipiravir (T-705), a broad spectrum inhibitor of viral RNA polymerase. Proc Jpn Acad Ser B Phys Biol Sci 93:449–63. 10.2183/pjab.93.027 PMC5713175 28769016

[CIT0020] Ghasemnejad-Berenji M, Pashapour S. (2021). Favipiravir and COVID-19: a simplified summary. Drug Res (Stuttg) 71:166–70. 33176367 10.1055/a-1296-7935 PMC8043595

[CIT0021] Hassanipour S, Arab-Zozani M, Amani B, et al. (2021). The efficacy and safety of Favipiravir in treatment of COVID-19: a systematic review and meta-analysis of clinical trials. Sci Rep 11:11022. 34040117 10.1038/s41598-021-90551-6 PMC8155021

[CIT0022] Jeon S, Ko M, Lee J, et al. (2020). Identification of antiviral drug candidates against SARS-CoV-2 from FDA-approved drugs. Antimicrob. Agents Chemother 64:1–9. 10.1128/AAC.00819-20 PMC7318052 32366720

[CIT0023] Joshi S, Parkar J, Ansari A, et al. (2021). Role of favipiravir in the treatment of COVID-19. Int J Infect Dis 102:501–8. 33130203 10.1016/j.ijid.2020.10.069 PMC7831863

[CIT0024] Kow CS, Ramachandram DS, Hasan SS. (2022). Future of antivirals in COVID-19: the case of favipiravir. Int Immunopharmacol 103:2021–3. 10.1016/j.intimp.2021.108455 PMC8666323 34959188

[CIT0025] Kumar GN, Jayanti VK, Johnson MK, et al. (2004). Metabolism and disposition of the HIV-1 protease inhibitor lopinavir (ABT-378) given in combination with ritonavir in rats, dogs, and humans. Pharm Res 21:1622–30. 15497688 10.1023/b:pham.0000041457.64638.8d

[CIT0026] Leiner S, Cipolla D, Eicher J, et al. (2017). Soft mist sprays. In: Hickey AJ, da Rocha SR, ed. Pharmaceutical inhalation aerosol technology. Boca Raton: CRC Press, vol. 35, 493–508.

[CIT0027] Marzouk HM, Rezk MR, Gouda AS, Abdel-Megied AM. (2022). A novel stability-indicating HPLC-DAD method for determination of favipiravir, a potential antiviral drug for COVID-19 treatment; application to degradation kinetic studies and in-vitro dissolution profiling. Microchem J 172: 106917. 34667334 10.1016/j.microc.2021.106917 PMC8518200

[CIT0028] Newman SP, Brown J, Steed KP, et al. (1998). Lung deposition of fenoterol and flunisolide delivered using a novel device for inhaled medicines * comparison of RESPIMAT with conventional metered-dose inhalers with and without spacer devices. Chest 113:957–63. 9554631 10.1378/chest.113.4.957

[CIT0029] Pilcer G, Amighi K. (2010). Formulation strategy and use of excipients in pulmonary drug delivery. Int J Pharm 392:1–19. 20223286 10.1016/j.ijpharm.2010.03.017

[CIT0030] Pitcairn G, Reader S, Pavia D, Newman S. (2005). Deposition of corticosteroid aerosol in the human lung by respimat® Soft Mist^TM^ inhaler compared to deposition by metered dose inhaler or by Turbuhaler® dry powder inhaler. J. Aerosol Med 18:264–72. 16181001 10.1089/jam.2005.18.264

[CIT0031] Sahin G, Halje P, Uzun S, et al. (2022). Antivirals and the potential benefits of orally inhaled drug administration in COVID-19 treatment. J Pharm Sci 16:861668. 10.1016/j.xphs.2022.06.004 PMC9181835 35691607

[CIT0032] Shannon A, Selisko B, Le NT, et al. (2020). Rapid incorporation of Favipiravir by the fast and permissive viral RNA polymerase complex results in SARS-CoV-2 lethal mutagenesis. Nat Commun 11:1–9. 32943628 10.1038/s41467-020-18463-z PMC7499305

[CIT0033] Shiraki K, Daikoku T. (2020). Favipiravir, an anti-influenza drug against life-threatening RNA virus infections. Pharmacol Ther 209:107512. 32097670 10.1016/j.pharmthera.2020.107512 PMC7102570

[CIT0034] Takayama K. (2020). In vitro and animal models for SARS-CoV-2 research. Trends Pharmacol Sci 41:513–17. 32553545 10.1016/j.tips.2020.05.005 PMC7260555

[CIT0035] Taşlı NP, Gönen ZB, Kırbaş OK, et al. (2022). Preclinical studies on convalescent human immune plasma-derived exosome: omics and antiviral properties to SARS-CoV-2. Front Immunol 13:824378–15. 35401544 10.3389/fimmu.2022.824378 PMC8987587

[CIT0036] Teng Z, Kuang X, Wang J, Zhang X. (2013). Real-time cell analysis – a new method for dynamic, quantitative measurement of infectious viruses and antiserum neutralizing activity. J Virol Methods 193:364–70. 23835032 10.1016/j.jviromet.2013.06.034

[CIT0037] Udwadia ZF, Singh P, Barkate H, et al. (2021). Efficacy and safety of favipiravir, an oral RNA-dependent RNA polymerase inhibitor, in mild-to-moderate COVID-19: a randomized, comparative, open-label, multicenter, phase 3 clinical trial. Int J Infect Dis 103:62–71. 33212256 10.1016/j.ijid.2020.11.142 PMC7668212

[CIT0038] Vemuri DK, Gundla R, Konduru N, et al. (2022). SARS-CoV-2) degradation impurities: Identification and route of degradation mechanism in the finished solid dosage form using LC/LC–MS method. Biomed Chromatogr 36:1–16. 10.1002/bmc.5363 PMC9073977 35292997

[CIT0039] Wang M, Cao R, Zhang L, et al. (2020). Remdesivir and chloroquine effectively inhibit the recently emerged novel coronavirus (2019-nCoV) in vitro. Cell Res 30:269–71. 32020029 10.1038/s41422-020-0282-0 PMC7054408

[CIT0040] Wang Y, Chen L. (2020). Lung tissue distribution of drugs as a key factor for COVID-19 treatment. Br J Pharmacol 177:4995–6. 32424836 10.1111/bph.15102 PMC7276739

[CIT0041] Wiersinga WJ, Rhodes A, Cheng AC, et al. (2020). Transmission, diagnosis, and treatment of coronavirus disease 2019 (COVID-19): a review. JAMA 324:782–93. 32648899 10.1001/jama.2020.12839

[CIT0042] Zhang R, Li Y, Zhang AL, et al. (2020). Identifying airborne transmission as the dominant route for the spread of COVID-19. Proc Natl Acad Sci USA 117:14857–63. 32527856 10.1073/pnas.2009637117 PMC7334447

[CIT0043] Zhu Y, Li J, Pang Z. (2021). Recent insights for the emerging COVID-19: drug discovery, therapeutic options and vaccine development. Asian J Pharm Sci 16:4–23. 32837565 10.1016/j.ajps.2020.06.001 PMC7335243

[CIT0044] Zost SJ, Gilchuk P, Chen RE, et al. (2020). Rapid isolation and profiling of a diverse panel of human monoclonal antibodies targeting the SARS-CoV-2 spike protein. Nat Med 26:1422–7. 32651581 10.1038/s41591-020-0998-x PMC8194108

